# Processing of complex traffic scenes for effective steering and collision avoidance: a perspective, from research into human control, on the challenges for sensor-based autonomous vehicles on urban roads

**DOI:** 10.3389/fpsyg.2024.1347309

**Published:** 2024-03-05

**Authors:** John P. Wann

**Affiliations:** Royal Holloway, University of London, Egham, United Kingdom

**Keywords:** self-driving, automobile, steering, collision, vision, neuroscience, cycling

## Abstract

An overview is provided of behavioral research into human steering and collision avoidance including the processing of optic flow, optical looming and the role of the human mobile gaze system. A consideration is then made of the issues that may occur for autonomous vehicles (AV) when they move from grid-type road networks into complex inner-city streets and interact with human drivers, pedestrians and cyclists. Comparisons between human processing and AV processing of these interactions are made. This raises issues as to whether AV control systems need to mimic human visual processing more closely and highlights the need for AV systems to develop a “theory of road users” that allows attribution of intent to other drivers, cyclists or pedestrians. Guidelines for the development of a “theory of road users” for AVs are suggested.

## 1 Optic flow and steering

### 1.1 Human steering

This perspective presents an evaluation of what might be learnt from observations of how human drivers cope with the task of driving, that could inform future developments of road based autonomous vehicles (AVs). It is the case that many locomotor animals show remarkable ability to control their trajectories and avoid or exploit collisions (e.g., with predators or prey), but they have evolved to do so within their respective niche (Gibson, 1966). Road networks are not a naturally occurring niche, they are a niche that humans have designed around the capabilities of the human perceptuo-motor system. A bird, or bat, or rodent are all highly skilled at trajectory control, but do not recognize the constraints or priorities presented by human designed road networks. So whereas other animals may usefully inform the design of AVs in flight, or in subterranean environments, this review focusses predominantly upon human processing during driving, within the constructed niche of urban roads, and also their interaction with other human road users.

Human behavioral research has confirmed that optic flow is the predominant source of information in human steering (Wann and Wilkie, 2004) and the human neural system is sensitive to optic expansion (Lee, 1976; Kaiser and Mowafy, 1993). When the optical axis is fixed then optic flow should provide a clear indication of the direction of travel either in the form of a focus of expansion (FoE) for a linear trajectory, or curved path-lines for a curved trajectory (Warren and Hannon, 1988). Considerable debate arouse as to whether optic flow could be used in this manner when humans move their eyes (Warren and Hannon, 1988; Wilkie and Wann, 2003). A natural behavior during steering is to “look where you want to go” and to fixate and track road zones that a driver wishes to move through (Wann and Land, 2000; Wann and Swapp, 2000, 2001). In fact, that gaze behavior is what is recommended in advance driving and motorcycling guides and for racing cyclists. This gaze behavior produces a discord between what is projected on the retina (the retinal flow field) and what is ‘projected’ on the windshield (the optic flow field). When gaze moves to track road zones the FoE no longer indicates locomotor heading, as the singularity in the retinal flow field is at the (moving) point of fixation and because of gaze rotation the retinal flow field can be curvilinear even when the ground trajectory is linear (Wilkie and Wann, 2003). There are, however, solutions to how humans might use this retinal flow field to judge their linear or curved trajectory (Wann and Swapp, 2000) and evidence that those changing patterns can be detected by the human neural system (Furlan et al., 2014). There is also strong evidence that the egocentric visual direction (EVD) of steering targets, detected either through head & gaze angle or using a frame of reference from the vehicle can be used either in combination with, or in place of, optic flow to control steering (Wann and Land, 2000; Wann and Swapp, 2001; Wilkie and Wann, 2002). The accumulated research supports the view that optic flow and FoE as originally defined by Gibson (1966) is not sufficient to guide human steering during high-speed travel on complex trajectories, but that humans use additional inputs such as gaze angle and EVD (Wilkie and Wann, 2002; Robertshaw and Wilkie, 2008). An interesting reflection on EVD is that it provides some of the information that is provided by a LIDAR system in an instrumented vehicle. In provides the angular orientation of an object of interest or hazard. What is less precise in human detection is the distance of that object. In principle that could be detected from vertical gaze angle, but that input changes with the inclination of the terrain and seated eye-height, whereas pictorial cues are unreliable and sensitivity to binocular information for distance declines rapidly over 10 m. So, unlike LIDAR, the human driver has limited ability to accurately estimate distance.

### 1.2 Automated vehicles and simple environments

Sensing for the steering of an automated vehicle (AV) would seem to be somewhat simplified. At first pass there is no need for the cameras to move and they can remain on a fixed axis, so there is no discord between optic flow and what is projected on the sensing surface of the camera. In addition, a LIDAR system can support the trajectory control system by detecting the angle and distance of objects in the forward scene and whether they might present a hazard for the planned trajectory. Furthermore, whereas a human driver turns a wheel, or leans a motorbike, and then detects the rate of turning from visual information to adjust their action, an automated system has direct access to the state of the steering system, the rate of turning and forward speed. Those combined inputs of external and internal information allow current (*circa* 2023) AVs to cope well in simple environments, such as straight roads with simple junctions and uncluttered freeways/motorways. Because human drivers are sometimes subject to distraction, inattention, undue haste, and emotional agitation, then AVs present a preferable transport solution for journeys through simple environments. But there are some issues that arise when the environment diverges from the simplicity of the freeway.

### 1.3 Automated vehicles and human drivers in complex environments

**Figure 1:**
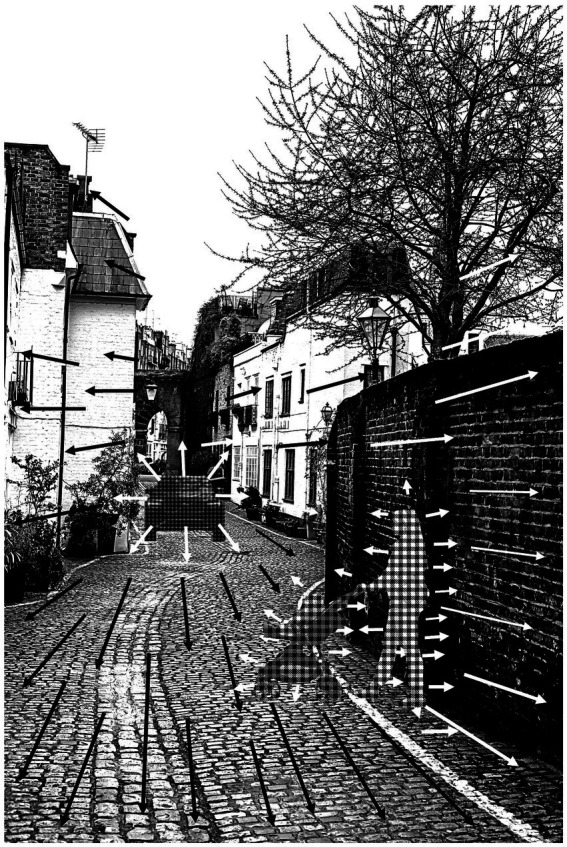
Optic flow fields in a complex driving environment. In traveling down an urban street a human driver or AV will be presented with an optical flow field from the expanding scene as a consequence of self-motion (illustrated by black arrows and white arrows on the dark wall). But embedded within this flow field is additional local expansion from an approaching vehicle (white arrows). There is also optical expansion of a pedestrian and lateral translation of that pedestrian + buggy moving into the road. To ensure safe passage the global flow field must be separated into these sub-components. (Image of “Thin Houses, London” reproduced under-license from iStockphoto.com).

Simple traffic environments, where the roads are predominantly straight, of constant lane width, and with geometrically simple junctions, typically take vehicles between or around dense urban centers. Once you enter an urban center that has evolved through historic iterations of building & expansion, then the situation can be much more complex. An AV needs to parse a visual scene to encode key details such as the permitted path and its width, any minor hazards such as a raised curb or a gutter, and any major hazards such as other vehicles (stationary or moving), bollards, cyclists, pedestrians (Figure 1). None of those features are accurately captured by geometric maps or satellite imagery. Maintaining a trajectory between two lane markings on a freeway is a task that an AV can complete better than a human driver. But upon entering a historic European city, road width can change unexpectedly, a raised curb can be replaced with a thin band of smooth surface at the edge of a cobbled street intended for pedestrians, and it is not uncommon for a street to be of insufficient width for 2 vehicles to pass, even though it is intended as a 2-way road (Figure 1). Add to that scene pedestrians moving transverse, often not using formal crossing points, but just using their own judgment of where there is a sufficient gap in the traffic to cross. This then raises the issue of AVs being able to make reliable assumption about human intention, which is discussed in Section 3. In summary, it is clear that AVs can complete the task of driving on freeway-type road systems without the human failings of distraction, impatience or fatigue. But environments such as displayed in Figure 1 present complex flow fields that merge vectors from self-motion, the motion of other vehicles and of pedestrians and road/pathway features that do not conform the standards of freeway-type road systems. To safely negotiate this type of environment without repeated freezing an AV guidance system needs to acquire a level of visual and cognitive processing that approaches the level of a human driver.

## 2 Anticipating collision

### 2.1 Human collision detection

One of the primary requirements of vehicle transport systems is that collision is avoided. Most visually guided animals have evolved to accurately judge imminent collision (Lee, 1976; Wang and Frost, 1992; Tammero and Dickinson, 2002). For predators, or in human sport, the aim might be a controlled collision (e.g., a football tackle, or a bird catching prey), but there are hardly any circumstances in vehicle use where a controlled collision is the intended outcome (the exception being highway police intercepts). The primary alerting signal for human drivers, and for other animals, appears to be optic expansion or ‘looming’. We have evidence that sub-cortical areas of the human brain are sensitive to looming (Billington et al., 2011), equivalent to what has been observed in other animals, such as pigeons (Wang and Frost, 1992). Those signals are then relayed to higher cortical areas that deal with spatial orientation and action (Field and Wann, 2005). This dorsal stream processing for potential action runs parallel to ventral systems that process object recognition (Goodale and David Milner, 1992; Goodale and David Milner, 2018). The two systems can operate over different time courses, such that a human may ‘instinctively’ raise their hand to protect their face from an approaching object and only realize a second or so later what the object was. There can be discrepancies in the other direction. In is possible for a driver to be sat in a queue of traffic fully aware of the make, color, model of the vehicles ahead, but unaware that their handbrake is not on and that they are slowly rolling forwards, because the looming signal is below the human threshold for detection. But in most road transport settings we can assume that the systems are operating over a similar time course and that a human driver recognizes objects or other road users within the same time window that s/he registers that they are approaching. That is important because it is the way in which a human driver parses the scenes such as shown in Figure 1, ventral processing providing object recognition and dorsal processing detecting threats or non-threats.

### 2.2 AV detection of potential collision in complex environments

If we consider the scene shown in Figure 1, it is important to recognize other vehicles and process their time to passage and that should be handled well in an AV by either a visual-template and/or LIDAR, but what does that same system process about the pedestrian? Current AV systems do recognize pedestrians, but how well do they process the low black object that projects ahead of the pedestrian. Clearly there is a surface that should be detected, but there are other surfaces that project into the path on the other side (planting tubs). In this narrow street it may be necessary to pass close to the planting tubs, but does the system have the capacity to appreciate that passing close to the child’s buggy is not acceptable. It is easy to take for granted the seamless integration of object recognition and collision detection in human processing and there can be false confidence taken from the performance of AVs on freeways. But on the freeway the task is just staying in lane while avoiding collision with the other metal boxes, a very simple processing task. Inner city areas are densely populated with much more complex shapes; adult pedestrians, children, upright cyclists, recumbent cyclists, children on bikes, pushchairs and shopping trollies, pedestrians pushing bikes, electric wheelchairs and the projected forms of these morph and change as they move and rotate. The argument is not that an AV/AI system would not be able to recognize each of those road users, but that the time-course of that object recognition needs to be seamlessly integrated with the collision processing system.

### 2.3 Detecting cyclists

A useful example is to consider that case of AVs and cyclists. Cars, trucks, motorcycles are rigid structures, they may re-orient, but they do not deform in their shape and there are orthogonal surfaces that should be readily detected by a ranging system. The label of “cyclist” is used to refer to a very heterogeneous cluster of road users. There are commuters who may be riding upright bikes, possibly with loose clothing that billows in the draft and presents a changing shape of soft-surfaces; there are cyclists clad in lycra, adopting aerodynamic positions with the aim that the body and bike present the smallest possible surface area; there are cyclists on recumbent bikes with a profile much closer to the ground; there are cyclists towing buggies that might contain children and the critical surface to avoid is not the upright figure of the cyclist, but the low cloth-clad canopy of the rear of the buggy; there are trikes; rickshaws; delivery cyclists with huge back-packs. All of these could be identified by a well-trained machine vision system, but what is the training set? How well does it capture the many variants of shape and form that cyclists can present? Cyclists are one of the most vulnerable road user groups, so safety is critical (Wegman et al., 2012). A collision speed that might result in a minor incident with another car could result in a critical injury for a cyclist. So if an AV is unable to find an ideal match for the moving object ahead of them does it follow 5 m behind a slow moving cyclist with a buggy for an extended period and cause congestion behind? There is also the time-frame over which identification needs to take place. Cars tend to conform to the carriageway markings, tend to follow one another, or approach on linear trajectories and their path over the next 5 s is generally predictable. Much to the chagrin of human drivers, cyclists can come from a wide range of places; from off the pavement, from side turnings, from behind the AV, they may have been occluded by another cyclist/vehicle ahead but then drift sideways to indicate a turn, they appear and disappear more readily than motorized vehicles. As a consequence the time frame for image identification can be very short. In general humans cope well with this type of short-term responsive processing, although ‘looked but failed to see’ accidents of cars, with human drivers, colliding with cyclists are far too high (Scholes et al., 2018). One advantageous feature that human drivers can employ is their mobile gaze system. Section 1.1 outlined that humans do ‘look where they want to go’ and that can present a retinal-flow problem to be solved at a cortical level. Section 1.2 then stated that at first pass there is no need for AV vision systems to move in the same way. But human mobile gaze also allows a driver to rapidly fixate objects with their central vision and resolve any ambiguities with the high resolution that the fovea affords. From the computational perspective, machine vision systems could be trained to recognize cyclists of different forms, recognize towed buggies, electric scooters, pushchairs traveling along a wide range of trajectories. Systems could sample over a sufficient time-frame to filter out shape deformations, they can also use those samples to extract ‘looming’ to supplement LIDAR distance/speed which may be less precise when the surfaces are small, rounded, soft and moving. But the issue is how comprehensive the classification is and the time-frame over which object classification is merged with collision-detection.

## 3 Reading human intention

### 3.1 The intention of other drivers

This is an area where there is a paucity of research, but observation of human drivers suggests that they make assumptions about the intentions of other drivers, even though those assumptions may sometimes be erroneous. If we return to considering freeway driving, most of the task is dictated by following other vehicles at a safe distance, responding to brake lights in the vehicles ahead, conforming to stop-signs, and there is little need to make assumptions regarding driver intention. There are some instances where an AV might infringe some unwritten rules. For example, if an AV system decides it is time to change lane on a freeway, it might confirm that although there is a vehicle approaching in the outside lane but that it is sufficiently distant and there is ample time to complete the lane change. But if that vehicle is approaching significantly faster than the AV, then the approaching human driver may have to apply the brake. The action of the AV was perfectly legitimate and safe, but if a human driver had executed it, the approaching driver would see that as an act of ‘blocking their progress’ and be annoyed, even though the approaching driver may have been traveling above the speed limit. This type of encounter between human drivers is quite common and can lead to dangerous behavior, by either driver. So, this raises a question; should an AV system also be able to make assumptions about whether action A or B might annoy a human driver who appears to be in a hurry? Once the AV is off the freeway and negotiating smaller European-type city streets, reading human intention becomes more important (Table 1). In most European towns and cities there are streets with pinch-points where there is insufficient space to pass an oncoming vehicle (Figure 1). Sometimes these are by design as a traffic calming measure, sometimes they are historic, such as a narrow bridge and sometimes they are caused by parked vehicles that have narrowed the passageway. In such cases it is usual for downstream and upstream drivers to take turns to wait or go. With just two vehicles there is typically just a slight slowing of one vehicle, maybe a wave of a hand, that indicates that one driver is conceding to the other. When there is a queue of vehicles in either direction there are unwritten conventions that after a driver has allowed 2 or 3 oncoming vehicles to pass, it is then their turn to move and the next oncoming vehicle should wait. The conventions probably vary geographically, and culturally, but infringing them can result in angry responses from other drivers. So what does an AV know about such conventions for acceding passage? Does an AV recognize that, in a narrow city street such as shown in Figure 1, following drivers might expect the lead vehicle to pass on oncoming vehicle with only a few centimeters of clearance in order to maintain progress, even though it would infringe upon the AVs standard safety protocol? There is also the issue of how human drivers respond to an oncoming AV at pinch-points. There is no animate-other, no eye contact or flexing of the hand to indicate “you come.” It is also the case that only a small hesitation in one driver can reverse the agreement. It seems unlikely that human drivers will grant passage to a driverless AV and at peak transport times an AV may be unduly delayed at a pinch-points, to the annoyance of following human drivers. One approach to this would be to teach an AV to drive assertively, but that in essence is teaching the system to mimic the poor behavior of impatient, aggressive human drivers, which does not seem a prudent direction.

**Table 1:** Guidelines for development of a theory of road users (ToRU) for autonomous vehicles.

Other road user	Category	Potential mental states of other road user	Signaling features and behaviors
Car, Truck, Motorcycle Driver	Optimal	Attentive, Unhurried, Calm, Considerate, Confident	Approach kinematics, Positioning, Gaze patterns, Gesture^i^, Age^ii^.
	Sub-Optimal	Distracted, Hurried, Assertive, Aggressive, Careless, Lacking-confidence	Approach kinematics, Positioning, Gaze patterns, Age.
Cyclist	Optimal	Experienced, Confident, Attentive, Aware, Cautious, Decisive	Speed, trajectory, lateral motion, gaze patterns (monitoring), direct non-verbal communication, body-language, Age, Appearance^iii^.
	Sub-Optimal	Inexperienced, Nervous, Indecisive, Over-confident, Aggressive, Injudicious	Speed, trajectory, lateral motion, gaze patterns, body-language, restricted audio (e.g., headphones), Age, Appearance.
Pedestrian	Optimal	Experienced, Attentive, Calm, Unhurried, Considerate, Confident	Age, walking speed & trajectory, Gaze patterns, Body Language.
	Sub-Optimal	Inexperienced, Distracted, Hurried, Careless, Lacking-confidence	Age, walking speed & trajectory, Gaze patterns, Body Language.

^i^Gestures are common between human drivers and/or pedestrians but it is unlikely that humans would gesture to an AV.

^ii^Age appears across all categories because it might be assumed that a young driver may behave differently from an elderly driver or a child pedestrian/cyclist differently from an experienced adult road user.

^iii^Appearance (apparel & type of bicycle) may not be reliable, but a cyclist in lycra-type clothing, wearing a helmet, on a “race-bike” may be indicative of a different pattern of riding from a cyclist in office-wear, without a helmet, riding an upright city-hire-bike.

### 3.2 The intention of pedestrians

In Section 2.2 I discussed the issue of identifying road users such as cyclists and pedestrians and classifying collision risk, but attentive human drivers are also able to make assumptions about the intentions of these other road users (Table 1). The majority of pedestrians are on the sidewalks. At regular periods some of them will approach the edge of the sidewalk with the intention of crossing the road. On observing this any vehicle, whether AV or human driver, could stop, but the convention is that pedestrians should avoid stepping directly in front of oncoming vehicles, so the vehicles continue while pedestrians wait at the side of the road. But there may be the case that a young child has stepped away from its parent, moves to the edge of the sidewalk, in which case an attentive human driver would slow and be ready to brake. An equivalent behavior could be set for an AV in response to objects classified as “child.” But an attentive driver can detect the subtle difference between “child holding parent’s hand” and “child not holding hand, child moving leg, parent on phone,” the general assumption of a human driver would be that only the second case raises the potential for emergency action. Let us consider other subtle cues to pedestrian behavior; An adult walks toward the edge of the sidewalk, they have ear buds in and are talking, they are staring at a phone screen and have not looked sideways down the road; An adult walks toward the edge of the sidewalk, but their gait is unsteady, they stand at the edge swaying for a moment as they look around but without seeming to fixate any oncoming vehicle. In both of those cases an attentive and careful human driver would elevate the risk assessment of the situation and be ready to take action should the pedestrian step into the road.

### 3.3 The intentions of cyclists

We have considered the potential difficulty in detecting and classifying cyclists, but there is also the need for drivers to make assumptions about the intention of cyclists. There is already evidence that in classifying cyclists human drivers may make assumptions about cyclist ability and give a wider berth to cyclists that they think are higher risk (Walker, 2007). Very few bicycles are equipped with turn-indicators and traditionally hand-signals are used by cyclists, but there are difficulties in using hand-signals. To signal a turn the cyclist needs to remove a hand from the bars and from one brake, and that might be the primary (front) brake. On uneven or crowded roads riding one-handed can be difficult. In addition, a cyclist does not want to stick their hand out and get it struck by a passing vehicle, so they are looking for a distance-time gap in which to indicate and maneuver, but they have poor visibility of the traffic behind. To an experienced human driver the key behavior that indicates that a cyclist intends to move in the lane is the act of them looking behind (Table 1). Respectful road sharing would then lead to the human driver slowing, allowing the cyclist to check back once more, indicate and then maneuver. If an AV classifies the cyclist correctly, judges that there is room to pass, but fails to code the cyclist’s intention on the basis of detecting the rear-ward glances then the AV is mimicking the behavior of too many drivers who fail to respect cycling as benefit to urban road use rather than an impediment (Fruhen and Flin, 2015; Fruhen et al., 2019).

### 3.4 The advantage of mobile gaze

Sections 3.1–3.3 outlined situations in which it is advantageous to guess at another road user’s intention. That is a cognitive process, but it is supported by accurate perception. Human drivers deploy their gaze to where they need to extract information. It might be to the apex of a tight bend (Land and Lee, 1994), it may be toward an oncoming driver or it may be toward a pedestrian or cyclist to resolve uncertainty or intent. High resolution foveal vision can allow a human driver to discern subtle patterns of movement, or a brief glance, by the other road user (Table 1). Fixations can be ~200 ms and using them a human can sample a number of details within a second to resolve ambiguities. It is difficult to see how a static machine vision system, even with multiple cameras, can match the performance of human gaze in resolving uncertainty and intent.

## 4 Summary

Control systems for AVs were initially developed using simple environments, where the steering requirements were either maintaining a straight path or staying within delineated bounds around a smooth curve. As a result they cope well with freeway-type road networks and cities that have been designed on a grid network with signaled intersections. They are also good at classifying time-to-collision with other vehicles, regulating speed, and do not suffer from the human frailties of distraction, inattention and emotional agitation. But that optimal performance on grid-type road systems among other solid metal vehicles can imbue a dangerous confidence in the ability of AVs to cope with all road environments.

Inner city streets that do not conform to a modern grid system can provide a more difficult challenge. Two delineated lanes can merge into a single passageway or pinch point where decisions as to ‘right of way’ may depend on regional convention and assumptions of other-driver intent. The simple optic-flow and optic-expansion (looming) encountered on the freeway is conflated with objects of myriad shape and form, some of which will loom, as a consequence of the AVs motion, but are static in the world, some of which will move transversely and change shape (pedestrians), some of which may be non-looming in a moving flow field, but changing shape (following a cyclist). A machine vision system can be trained to recognize all these objects and discern their motion path. But the issue is how broad the training and how comprehensive the classification. The guidance systems of an AV cannot ‘evolve’ at the cost of hard interactions with soft road users.

In this respect the human visual system has a considerable advantage. It has evolved to cope with a very variable environment and can classify a running rabbit as readily as a car, motorcycle or cyclist. Alongside this ‘ventral’ classification system is a ‘dorsal system’ that fast-tracks perception-for-action. The two systems work together because that was essential for species survival. What is fortuitous is that a locomotor control system that evolved to cope with motion up to ~20kph (fast human running) appears to allow humans to control vehicles at 5-times that speed. It is conceivable that the human neural systems that process optic motion vectors (e.g., cortical area MT & MST: Smith et al., 2006) could have failed to cope with the vectors that arise from high-speed driving.

A further advantage that the human driver takes onto the road arises from evolution in social settings. One of the features that has enhanced human interaction is the ability to discern what another person’s intention might be. In cognitive psychology this would typically be labeled Theory of Mind (Frith and Frith, 2005; Yang et al., 2015), but it can also be captured within the Theory of Affordances (Gibson, 1986; Ch8). Within the latter framework we recognize that certain properties of the behavior of the other (person/animal) transmit information that they may act in a particular manner. In truth this can only ever be the reflection of surface behavior, there is no insight into the actual thought of the other party, but for effective road usage a simple set of assumptions about the behavior of others may suffice. For this we could posit that AVs would benefit from a “Theory of Road Users” which is based upon observable patterns of dynamic behavior and as such lies closer to the Theory of Affordances than the Theory of Mind.

I have outlined in Section 3 a selection of situations where inferring intent to the other road user is necessary to avoid congestion or in some cases may be critical to maintain safety. In the latter case it is the most vulnerable road users such as pedestrians and cyclists that are most likely to be placed at risk by AVs with no “theory of road users.” Table 1 presents a categorization of some of the judgments that humans may make about other road users and the features or behaviors that may support those judgment, with that caveat that those assumptions may sometimes be erroneous. There have been neural network systems developed that attempt to predict driver behavior or intent, but a number of these are using vehicle kinematics to estimate trajectories (Bonyani et al., 2015; Girma et al., 2020), but not yet attempting the broader process of detecting potential behavior of pedestrians and cyclists, which remains a significant challenge (Gilpin, 2021). What would be valuable would be the identification of the most reliable dynamic features of road user behavior that can lead to computational models arriving at robust estimates of emergent behavior (e.g., Markkula et al. (2023)).

Looking forwards over the next decade, the road situation would be eased considerably if AVs were the only vehicles on the road, but estimates as to when that might be the case extend to 2050. So, over the next decade AVs need to evolve to cope with interaction with human drivers. Interactions with pedestrians and cyclists are likely to continue as the health benefits of cycling and walking are part of a global green transport agenda. Hopefully the next decade sees more people walking and cycling to work or school and less vehicles in cities, irrespective of whether they are EV-AVs or not. One approach is to try and separate cyclists from motorized vehicles, but this is only partially feasible in most major cities, so designers of AV systems should assume that cyclists, in all their myriad forms, will continue to be present on roadways in increasing numbers. Given the current status of AV development in 2023, I would not be happy being followed by an AV while cycling. Neither would I be relaxed if I was a passive driver in an AV that was approaching some cyclists, I would prefer to assert human control and rely upon 20 years of road experience, plus ~6million years of evolution.

## Data availability statement

The original contributions presented in the study are included in the article/supplementary material, further inquiries can be directed to the corresponding author.

## Author contributions

JW: Writing – original draft.
